# Case-matched study of short-term effects of 3D vs 2D laparoscopic radical resection of rectal cancer

**DOI:** 10.1186/s12957-017-1247-8

**Published:** 2017-09-22

**Authors:** QingMin Zeng, Fuming Lei, ZhaoYa Gao, YanZhao Wang, Qing Kun Gao

**Affiliations:** General Surgery, Gastrointestinal Surgery, Peking University Shougang Hospital, Jin Yuan Zhuang Road No. 9, Beijing, 100041 China

**Keywords:** Three-dimensional, Stereopsis, Laparoscopy, Rectal cancer, Surgery

## Abstract

**Background:**

The purpose of this study is to compare and evaluate the security and efficacy of 3D vs 2D laparoscopy in rectal cancer treatment.

**Methods:**

Forty-six patients who suffered from rectal cancer and went on laparoscopic radical resection of rectal carcinoma in Peking University Shougang Hospital from Feb. 2015 to Mar. 2016 were included in the study. They were randomly divided into two groups. The 23 patients operated with the 3D system were compared with 23 patients operated with the 2D system by perioperative data.

**Results:**

There were no significant differences in age, sex, pathological type, tumor differentiation, TNM staging, and surgical procedures (*P* > 0.05). The average operating time of 3D laparoscopic surgery group (172.2 ± 27.5 min) was shorter than that of 2D group (192.6 ± 22.3) (*P* < 0.05); the rate of transfer to laparotomy is lower in 2D group (72.7%) than in 3D group (86.4%), but they have no significant difference; and the intraoperative blood loss (247.0 ± 173.6 ml vs 282.6 ± 195.6 ml), postoperative passage of flatus (2.8 ± 0.8 days vs 3.1 ± 1.0 days), and indwelling catheter time (5.6 ± 1.9 days vs 6.3 ± 2.0 days) in 3D group and 2D group (*P* > 0.05) were not significantly different. There were no differences in other complications between the two groups. No significantly different recrudescence and death rates were found between the two groups (*P* > 0.05).

**Conclusion:**

The 3D laparoscopy shortens the operation time of rectum cancer. 3D laparoscopic surgery is more efficient in treatment of rectal cancer than 2D laparoscopy and is worth of being generalized.

## Background

Since the first multicenter retrospective study was undertaken by Falk et al. in 1993 [1], laparoscopy in colon and rectal surgery has been proved to have lower complication rates and perioperative morbidity, shorter operation duration and hospital stay, and less postoperative pain and hospital cost compared with open surgery [2, 3]. However, surgeons actually work in a three-dimensional space but are guided in two-dimensional (2D) images provided by laparoscopy cameras which results in losing true depth perception and lacking spatial orientation that potentially increases the risk of errors and the operative time, and these limitations require more learning period and operation skills for surgeons [4].

The development of three-dimensional (3D) high-definition laparoscopy is offering the surgeons clearer depth of field and is developed as an alternative. Some studies have reported that 3D laparoscopy reduces the performance errors and operative duration [5] and also improves performance for surgical novices compared with 2D laparoscopy [6–8]. Two-dimensional laparoscopic radical resection of rectal carcinoma (LRRRC) has been performed for years; however, as the 3D technology was introduced, not any study has been conducted to compare the outcomings and differences between 3D and 2D LRRRC.

To address the issue whether 3D system offers better security and efficacy to LRRRC, we retrospectively analyzed two consecutive series of LRRRC performed by a single experienced surgeon with 2D and 3D systems, respectively.

## Methods

### Patients

A total number of 46 patients diagnosed with rectal cancer (T2-T3) were included in this study during the period between February 2015 and March 2016. Patients with locally advanced and/or distant metastasis or recurrence of rectal cancer were excluded. All the patients were assigned to receive either a 2D or 3D LRRRC by a single experienced laparoscopic surgeon, and general physical conditions as well as carcinoma stages of patients between both groups were well-matched. All the surgeries including 2D and 3D LRRRC were performed by a single experienced surgeon who was familiar with the 3D imaging system and both the 2D and 3D LRRRC procedures. All the surgeries were meanwhile performed by the other constant surgeon who acted as an assistant and camera operator. Patients with the distance ≥ 4 cm from bottom margin of cancer to anus received the modus operandi of anterior resection (AR), while others received the modus operandi abdominoperineal resection (ARP). Our research was performed in accordance with the principles outlined in the Declaration of Helsinki and was approved by the Medical Ethics Committee of the Peking University Shougang Hospital.

### Laparoscopic radical resection of rectal carcinoma

Under general anesthesia, the patients were placed in a lithotomy position and tilted on right lateral by 20°. A urinary catheter was inserted in order to avoid bladder injury. Pneumoperitoneum was created by vertical supraumbilical incision on the omphalos. Other ports were then positioned under direct vision in the right lower rectus abdominis (10 mm), right upper rectus abdominis (5 mm), and left lower rectus abdominis (5 mm). A fifth trocar (5 mm) would be added in the left upper rectus abdominis if necessary. After creating pneumoperitoneum and inserting the access ports, preliminary laparoscopy was performed to determine the margin of resection. Then, the colon sigmoideum was identified by a medial approach, and the superior rectal artery or inferior mesenteric artery was ligated. For the modus operandi of AR, the mesorectum was isolated under the bottom margin of cancer by 2~3 cm, and then, colon-rectum anastomosis was reconstructed using anastomat. For the modus operandi of ARP, the mesorectum was isolated to the lower level of the anococcygeal ligament and seminal vesicle (or posterior of the vagina in female); subsequently, resection of rectal cancer was performed. All the operations were performed by a single experienced group.

### Statistical analysis

All the data were presented with mean ± standard (*x* ± *s*) error. Differences between the groups were analyzed using Student’s *t* test. Differences in the distribution of nominal parameters were analyzed with *χ* ^2^ test. All the statistical analyses were performed with SPSS statistical package 17.0 (SPSS Inc., Chicago, USA), and *P* < 0.05 was considered to be statistically significant.

## Results

### Patients’ clinical parameters

As shown in Table 1, two groups were equal in enrolled subjects (23 subjects), and both were similar for gender (14 males and 9 females in 3D group vs 16 and 7 in 2D group), pathological type (18 adenocarcinoma and 5 mucinous adenocarcinoma in 3D group vs 13 and 10 in 2D group), cancer cell differentiated level (13 poorly to moderately-poorly differentiated and 10 moderately-well to well-differentiated level in 3D group vs 10 and 13 in 2D groups), and T-stage (4 T2 and 19 T3 in 3D vs 4 T2 and 18 T3 in 2D group). No significant differences of conversion to open surgery rate and various modus operandi were found between 3D and 2D groups.

**Table 1:** Clinical parameters in 3D and 2D groups

	3D group	2D group	*χ* ^2^	*P*
	(*n* = 23)	(*n* = 23)		
Gender				
Male	14(60.9)	16(69.6)	0.383	0.536
Female	9(39.1)	7(30.4)		
Pathology				
Adenocarcinoma	18(78.3)	13(87)	2.473	0.116
Mucinous adenocarcinoma	5(21.7)	10(13)		
Differentiation				
Poorly to moderately-poorly	13(56.5)	10(43.5)	0.723	0.376
Moderately-well to well	10(43.5)	13(54.5)		
T-stage				
T_2_	4(17.4)	4(17.4)	0	1
T_3_	19(82.6)	19(82.6)		
Conversion to open surgery				
No	3(13.6)	5(27.3)	0.605	0.437
Yes	20(86.4)	18(72.7)		
Modus operandi				
AR	16(69.6)	17(73.9)	0.107	0.743
ARP	7(31.4)	6(26.1)		

*2D* two-dimensional, *3D* three-dimensional, *AR* anterior resection, *APR* abdominoperineal resection

### Operative parameters

Table 2 shows the comparison of operative parameters between 3D and 2D groups. The operation duration is significantly shorter in the 3D group (172.2 ± 27.5 min) than in 2D group (192.6 ± 22.3 min, *P* = 0.01). However, no significant differences were found in operation hemorrhage, duration of retention catheterization, amount of lymph node detection, flatus time, and duration of hospitalization (*P* > 0.05).

**Table 2:** Operative parameters in 3D and 2D groups

Parameters	3D group (*n* = 23)	2D group (*n* = 23)	*t*/*χ* ^2^	*P*
Operation duration(min)	172.2 ± 27.5	192.6 ± 22.3	2.079	0.008
Operation hemorrhage(min)	247.0 ± 173.6	282.6 ± 195.6	0.204	0.654
Duration of retention catheterization(day)	5.6 ± 1.9	6.3 ± 2.0	0.017	0.897
Lymph node detection	17.3 ± 5.2	17.1 ± 5.3	0.264	0.610
Flatus time(day)	2.8 ± 0.8	3.1 ± 1.0	1.45	0.235
Duration of hospitalization(day)	11.5 ± 4.7	11.3 ± 3.65	1.93	0.633

*2D* two-dimensional, *3D* three-dimensional

### Complications

Complications including intestinal obstruction, anastomotic fistula, retention of urine, and sexual dysfunction were only observed in patients who received AR; one case of pulmonary infection and one wound infection were observed in patients who received ARP. No any significantly different complication rates were found between patients of the 3D and 2D groups (*P* > 0.05) (Table 3).

**Table 3:** Complications between 3D and 2D groups

Group	*n*	Pulmonary infection	Wound infection	Intestinal obstruction	Anastomotic fistula	Retention of urine	Sexual dysfunction	Complication rate
3D	23	2	2	1	2	1	1	8
2D	23	2	1	2	0	3	2	10
*χ* ^2^	–	0.000	0.357	0.357	2.091	1.100	0.357	0.365
*P*	–	1.000	0.550	0.550	0.148	0.295	0.550	0.546

*2D* two-dimensional, *3D* three-dimensional

### Follow-up

All the patients were followed up for 5 to 17 months (11.24 ± 3.20); eight cases of recrudescence (34.8%) and two cases of death (8.7%) were observed in the 3D group, compared with seven (30.4%) and two (8.7%) cases, respectively, in the 2D group. No significantly different recrudescence and death rates were found between the two groups (*P* > 0.05).

## Discussion

The loss of spatial depth information in a 2D imaging system is a great challenge for surgeons who need to operate within the 3D scene but can only observe on 2D display. This requires high hand-eye coordination skill and good cooperation between surgeons and assistants [9, 10]. The latest 3D imaging systems are a dual-lens system; two separate lenses with two cameras are present within a single laparoscope. Respective images are captured by each camera, then displayed and synchronized on the monitor [11]. This most recent 3D vision model offers superior quality of images and stereoscopic vision for surgeons which can be treated as an alternative to conventional 2D imaging so that it overcomes the shortcomings of 2D laparoscopic surgery [12].

Increasing studies have been indicating that less time was needed for radical resection of rectal cancer performed with 3D laparoscopic surgery than 2D [13, 14]. Surgeons who performed with the 3D system experienced as good depth and spatial perception as in the open surgery compared with 2D system [15]. Due to the better spatial vision and high-definition images in the 3D system, adjacent organs could be easy to recognize, and also, the possibilities of wound and hemorrhage in operation were reduced, which offer the basis of shorter post-surgery recovery duration. The comparative study of 3D and 2D laparoscopic surgery in gastrointestinal tumors has been performed, which has demonstrated that 3D laparoscopic surgery can improve the spatial location and depth of operation, decrease the difficulty of fine operation, and shorten the operation time. Previous study has reported that significant shorter operation duration and less hemorrhage were observed in 3D laparoscopic surgery compared with 2D [16]. In this study, we also found significant shorter operation duration in 3D group, which was consistent with the previous study. However, as to the hemorrhage in operation, we failed to find any significant difference. In our study, conversions to open surgery were performed to a few patients in both groups, and the different experiences, skills, and abilities between surgeons in various studies would also influence the final results. We hypothesized these factors would contribute to the inconsistent results between different studies.

During the LRRRC, the 3D system offers clearer anatomic structure views of the pelvic floor so that it increases nerve protection and reduces the risk of wound in the male seminal vesicle and female posterior vaginal wall. Steric and enthesis of the musculi levator ani could be clearly identified which reduced operation difficulty of TME [17]. The high definition of cameras and monitors also provides accurate views on fine structures of organs in the pelvic, so, that decreased the possibilities of pelvic plexus wounds during operation and the associated postoperative complications. Some studies have reported the 3D system required a significant shorter learning curve [10, 18, 19]. In this study, the operation duration in 3D groups was 172.7 ± 28.0 min, which was significantly shorter than that in 2D group 192.7 ± 22.8 min (*P* = 0.010); our result was similar to the result from Vimalraj Velayutham et al. who reported a significant shorter operation duration for 3D Laparoscopic liver resection (225 ± 109 min) compared with 2D (192.7 ± 22.8 min), (*P* < 0.05). Later studies conducted by Kinoshita et al. [14] and Currò et al. [11] also found similar results. The stereo and high-definition images from the 3D system offer a good depth perception and clear anatomic structures for the surgeon who might be responsible for the shorter operation duration in 3D surgery. Although not any significant difference was observed in other parameters between the two groups in our study, the absolute values in 3D group were higher than those in 2D group; larger sample would be needed to detect more advantages of 3D system.

Although 3D technology was introduced in the late of twentieth century, this system is not yet standard in most hospitals because of side effects and some previous controversial conclusions. Side effects such as eye strain, headaches, dizziness, and physical discomfort in over-depth perception and color distortion in monitor, were especially more serious in the condition of organs staining with blood [20]. Studies have reported that the 3D system reduced the learning curve; however, most of the studied samples included in this study were novice surgeons [11, 21, 22]; in fact, for those skilled laparoscopic surgeons, the difference of performance between 3D and 2D laparoscopic surgeries differ little. In our study, we speculated that the reason why the 3D group had a significantly shorter operation duration was because both 3D and 2D LRRRC were applied in our hospital in the same period; the 3D system offered the surgery more accurate performance, so, that reduced the time. This advantage might be fading as the surgeon accumulates his operative skills and experiences. More samples and studies are needed to confirm our speculation. There were also some limitations in our study. The number of the included patients in our study is small. The follow-up time is relatively short. Further research with larger sample size and longer follow-up time is needed.

## Conclusion

In conclusion, on the basis of our results, the 3D system significantly reduced the performance time of LRRRC; although the 3D system offers better depth perception, the incidence rates of post-surgery complications differed very little. Further comparative studies are required to clarify the actual advantages of 3D system in LRRRC and to verify our results.

## Abbreviations

AR: Anterior resection
ARP: Abdominoperineal resection
LRRRC: Laparoscopic radical resection of rectal carcinoma
